# Impact of tissue-independent positron range correction on [^68^Ga]Ga-DOTATOC and [^68^Ga]Ga-PSMA PET image reconstructions: a patient data study

**DOI:** 10.1007/s00259-024-07061-6

**Published:** 2025-01-29

**Authors:** Prodromos Gavriilidis, Felix M. Mottaghy, Michel Koole, Tineke van de Weijer, Cristina Mitea, Jochem A. J. van der Pol, Thiemo J. A. van Nijnatten, Floris P. Jansen, Roel Wierts

**Affiliations:** 1 Department of Radiology and Nuclear Medicine, Maastricht University Medical Center, Maastricht, The Netherlands; 2 Research Institute for Oncology and Reproduction (GROW), Maastricht University, Maastricht, The Netherlands; 3 Nuclear Medicine and Molecular Imaging, Department of Imaging & Pathology, KU Leuven, Louvain, Belgium; 4 Department of Nuclear Medicine, RWTH University Hospital, Aachen, Germany; 5 Molecular Imaging, GE HealthCare, Waukesha, WI USA; 6 Research Institute of Nutrition Translational Research in Metabolism (NUTRIM), Maastricht University, Maastricht, The Netherlands; 7 Cardiovascular Research Institute Maastricht (CARIM), Maastricht University, Maastricht, The Netherlands

**Keywords:** Positron Emission Tomography, Gallium-68, Positron Range, Positron Range Correction

## Abstract

**Purpose:**

The positron range effect can impair PET image quality of Gallium-68 (^68^Ga). A positron range correction (PRC) can be applied to reduce this effect. In this study, the effect of a tissue-independent PRC for ^68^Ga was investigated on patient data.

**Methods:**

PET/CT data (40 patients: [^68^Ga]Ga-DOTATOC or [^68^Ga]Ga-PSMA) were reconstructed using Q.Clear reconstruction algorithm. Two reconstructions were performed per patient, Q.Clear with and without PRC. SUV_max_ and contrast-to-noise ratio (CNR) values per lesion were compared between PRC and non-PRC images. Five experienced nuclear medicine physicians reviewed the images and chose the preferred reconstruction based on the image quality, lesion detectability, and diagnostic confidence.

**Results:**

A total of 155 lesions were identified. The PRC resulted in statistically significant increase of the SUV_max_ and CNR for soft tissue lesions (6.4%, p < 0.001; 8.6%, p < 0.001), bone lesions (14.6%, p < 0.001; 12.5%, p < 0.001), and lung lesions (3.6%, p = 0.010; 6.3%, p = 0.001). This effect was most prominent in small lesions (SUV_max_: 12.0%, p < 0.001, and CNR: 13.0%, p < 0.001). Similar or better image quality, lesion detectability, and diagnostic confidence was achieved in PRC images compared to the non-PRC images as those assessed by the expert readers.

**Conclusions:**

A tissue-independent PRC increased the SUV_max_ and CNR in soft tissue, bone, and lung lesions with a larger effect for the small lesions. Visual assessment demonstrated similar or better image quality, lesion detectability, and diagnostic confidence in PRC images compared to the non-PRC images.

**Supplementary Information:**

The online version contains supplementary material available at 10.1007/s00259-024-07061-6.

## Introduction

Positron emission tomography (PET) is a nuclear medicine imaging modality using a radiotracer consisting of a chemical molecule coupled to a positron emitting radionuclide that is administered to the patient. The distance traveled by the positron before annihilation is called the positron range and depends on the kinetic energy of the positron as well as the density of the adjacent tissue [1–5]. It was demonstrated in the literature that positron range can have a negative impact on the image quality of PET images [1, 3–8]. The severity of this effect depends on the kinetic energy of the emitted positrons and thus on the radionuclide [1, 3–8]. The most used radionuclide in PET, Fluorine-18 (^18^F), features a mean positron energy of 252 keV (maximum energy: 635 keV) [9], resulting in negligible image degradation due to small positron range (mean positron range of ^18^F in water: ~ 0.4 mm [10]). In contrast, Gallium-68 (^68^Ga), features a higher mean positron energy of 844 keV (maximum energy; 1899 keV) [9], which results in greater image degradation [1, 3–5, 8].

Nevertheless, the growing number of clinically used ^68^Ga-labeled radiotracers [11–15] in combination with the commercial availability of ^68^Ge/^68^Ga generators has resulted in a high clinical use of ^68^Ga-labeled tracers in PET imaging. Furthermore, the clinical implementation of new promising ^68^Ga-labeled radiotracers, such as [^68^Ga]Ga-FAPI, may further increase the clinical use of ^68^Ga-PET imaging in the future [15]. Additionally, the intrinsic spatial resolution of new generation PET scanners will continue improving [16–18] and become comparable to the mean positron range of ^68^Ga (mean positron range of ^68^Ga in water: ~ 2.2 mm [10]). Hence, the contribution of the ^68^Ga positron range to the reconstructed PET resolution will become more prominent [19]. Therefore, it is important to consider a ^68^Ga-specific positron range correction to ensure a ^68^Ga-PET image quality and quantification in line with the image quality and quantification that can be achieved with ^18^F-PET.

Various techniques applied during or post-reconstruction to correct for ^68^Ga positron range effect have demonstrated potential improvements in image quality using both real and simulated phantom data [10, 20–25]. Positron range correction (PRC) was applied also via a deep learning model trained using simulated ^68^Ga mice model data as input and corresponding simulated ^18^F data as output [26]. This method reportedly enhanced image quality to a level comparable to ^18^F imaging [26]. In a separate study, the application of PRC during the iterative reconstruction resulted in improved image quality in live mice with neuroendocrine tumors [27]. Preliminary patient results also supported these improvements in image quality [10]. However, a larger cohort of patients and a greater number of lesions are required to confirm the potential clinical benefit of the application of a PRC in patient data.

The aim of this study is to explore the effect of a tissue-independent ^68^Ga PRC in [^68^Ga]Ga-DOTATOC or [^68^Ga]Ga-PSMA PET patient data through a head-to-head comparison of reconstructions with and without applying this ^68^Ga-specific PRC. To the best of our knowledge, this is the first study investigated the effect of tissue-independent PRC in a cohort of patient data.

## Materials and methods

### Patient data and acquisition protocol

All patients receiving a [^68^Ga]Ga-PSMA or [^68^Ga]Ga-DOTATOC PET/CT examination as part of standard care between October 2022 and July 2023, who were identified with at least one lesion showing radiotracer uptake, were included in this study. This resulted in a total of 40 patients (median weight: 81 kg, range: 51–118 kg) receiving [^68^Ga]Ga-PSMA (11/40) or [^68^Ga]Ga-DOTATOC (29/40). Prior to radiotracer administration, patients provided informed consent. Approval for this study was granted by the medical ethics committee METC azM/UM, Maastricht, The Netherlands (Date: 09-July-2021, No: 2021–2795). For [^68^Ga]Ga-PSMA PET imaging, patients were injected with furosemide (10 mg) intravenously before the tracer administration. The activity dosing for [^68^Ga]Ga-PSMA was 1.5 MBq/kg and the scan was performed 45 min post injection using a scanning time of 3 min per bed position. Before start of the PET acquisition, patients were asked to void. [^68^Ga]Ga-DOTATOC patients also received an activity dosing of 1.5 MBq/kg. However, PET acquisition started 30 min after tracer administration and consisted of 4 min per bed position. Prior to PET acquisition, a low-dose computed tomography (low-dose CT) was performed with a tube voltage of 120 kV, automatic tube current modulation ranging from 10 to 60 mA, and a noise index of 50%. The rotation time was 0.5 s, and the pitch factor was 0.984. All the patients were scanned with the Discovery MI LightBurst Digital 5-Ring PET/CT system (GE HealthCare, United States).

### PET reconstructions

PET patient data were reconstructed offline employing the Duetto PET reconstruction toolbox (version 02.18), which is a research software package provided by GE HealthCare. The voxel dimensions were 2.73 × 2.73 × 2.80 mm^3^. Q.Clear was the selected reconstruction algorithm. Q.Clear employs a noise regularization term, denoted as β, and uses the Block Sequential Regularized Expectation Maximization as optimizer [28]. For each patient, two image reconstructions were performed, one with PRC and one without PRC. For the PRC reconstructions the tissue-independent PRC for ^68^Ga presented in [10] was employed. Specifically, the positron range distribution profile of ^68^Ga in water was obtained via Monte Carlo simulation. That profile was then mapped into a 3D kernel which was applied as an additional image-based convolution term before the forward projection and after the backward projection steps of the iterative reconstruction. Further details are described in [10]. Additionally, both Time-of-Flight (ToF) information and Point-Spread-Function (PSF) modeling were incorporated, and standard vendor corrections for radioactive decay, scatter, randoms, normalization, attenuation, and dead time were applied in all reconstructions.

Previous studies demonstrated that implementation of a PRC during both the forward and backward projection steps of the iterative reconstruction can reduce image noise levels [10, 24]. Therefore, to obtain a fair comparison between the corrected and non-corrected images, β values were selected such that similar noise levels could be achieved between the corresponding corrected and non-corrected cases as explained in [10]. This resulted in a β value of 700 for the non-PRC reconstructions as used in clinical routine, and a β value of 600 for the PRC reconstructions, with the latter value derived from phantom measurements [10].

### Analysis

#### Lesion delineation and characterization

**Fig. 1:**
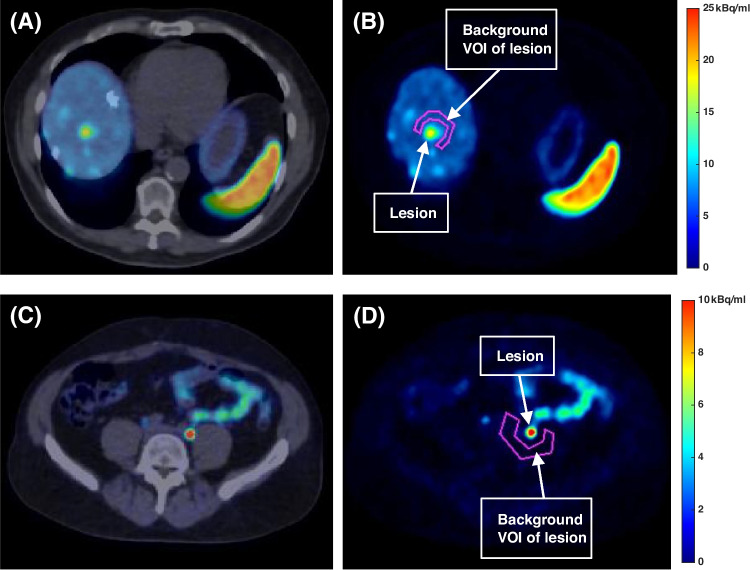
Examples of cross-sections of lesions from the fusion of PET/CT and the corresponding background VOIs. An example of lesion located inside a background with a relatively high activity background concentration (i.e. liver): **(A)** PET/CT fusion image, **(B)** background VOI of the lesion in the PET image. Example of lesion with a low background activity concentration: **(C)** PET/CT fusion image, **(D)** PET image with the background VOI of the lesion

Suspected lesions were identified by a trained nuclear medicine physician (F.M.) in the non-PRC reconstructions. The lesions were delineated using the software PMOD 3.7 (PMOD Technologies LLC, Switzerland). More specifically, a volume of interest (VOI) was created for each lesion based on a 50% isocontour threshold of the maximum voxel value within the VOI without background correction. Based on its volume, each lesion was as small (volume ≤ 1 cm^3^), medium (1 cm^3^ < volume ≤ 10 cm^3^) or large (volume > 10 cm^3^). Additionally, the lesions were also identified as soft tissue, bone or lung lesions. To avoid potential bias from patients with multiple lesions, a maximum of the 5 largest lesions per tissue type per patient was selected. Typical distance between the border of a lesion and the border of the background VOI was about 0.5 cm (see Fig. 1). Additionally, each background VOI was set to have a volume of at least 10 cm^3^. All the VOIs were initially generated in the reconstructions with PRC and then mapped onto the corresponding non-PRC reconstructions.

#### Quantitative analysis

To validate in patient data the phantom-derived β value of 600 for the PRC reconstructions, a spherical VOI with a radius of 10 mm was created in a homogeneous part of the liver and noise levels were calculated for both the PRC and non-PRC reconstructions. Liver was chosen for this comparison as it typically shows homogeneous radiotracer uptake. Moreover, in the presence of liver lesions, the VOI in the liver was created to not include any lesions nor being near lesions. The noise level was determined as the coefficient of variance (COV):

1 $$\mathrm{COV}=\frac{{\mathrm{SD}}_{\mathrm{Liver}}}{{\mathrm{Mean}}_{\mathrm{Liver}}}\cdot100\%$$

where ${\text{SD}}_{\text{Liver}}$ and ${\text{Mean}}_{\text{Liver}}$ were the measured standard deviation and mean activity concentration of the VOI in the liver.

To quantify the effect of the PRC on the image quality, the maximum standardized uptake value (SUV_max_) and the contrast-to-noise ratio (CNR) were obtained per lesion. Additionally, for each lesion, the mean standardized uptake value (SUV_mean_) and the peak standardized uptake value (SUV_peak_) derived from a 1 mL sphere with the highest uptake were also measured. The CNR of each lesion was calculated as:

2 $$\mathrm{CNR}=\frac{\mathrm{Mean}-{\mathrm{Mean}}_{\mathrm{Background}}}{{\mathrm{SD}}_{\mathrm{Background}}}$$

with $\text{Mean}$ and ${\text{Mean}}_{\text{Background}}$ being the measured mean lesion and background activity concentrations, respectively, while ${\text{SD}}_{\text{Background}}$ represents the measured standard deviation of the background activity concentration of that lesion.

#### Qualitative analysis

The reconstructed images were reviewed by five experienced nuclear medicine physicians (F.M., C.M., J.P., T.W., T.N.) with 4 to 27 years of clinical experience in the field. Both the PRC and non-PRC images were simultaneously presented per patient to the reviewers. Images were presented in random order and reviewers were blinded to the reconstruction settings. Moreover, reviewers were assigned to select the preferable image or report similar preference for each of the criteria: 1) image quality, 2) lesion detectability, and 3) diagnostic confidence. The reviewers performed the visual assessment independently with no interaction between each other until the scoring process was finished.

#### Statistical analysis

The statistical analyses were performed in R (R version 4.3.2). The percentage change was calculated of the PRC against the non-PRC reconstructions for the noise level (COV), SUV_max_ and CNR. These percentage changes were then tested for normality using the D’Agostino-Pearson omnibus normality test. Since deviation from normal distribution was observed for some metrics, the Wilcoxon signed-rank test was used to test for significance. To evaluate the degree of agreement between the reviewers’ visual assessment, the Gwet’s agreement coefficient (Gwet’s AC) [29] was calculated using two categories for each of the previously mentioned criteria. The first category included cases where the PRC images were scored as similar to or better than the non-PRC images for the corresponding criterion. In the second category, the non-PRC images were scored as better than the PRC images. Gwet’s AC was selected because skewed category distributions were expected. Although kappa (κ) statistics, such as Cohen’s kappa, are more commonly used, they produce low kappa values in cases with skewed category distribution (high prevalence) [29–31]. In such instances, Gwet’s AC is more stable [29–31] and is thus typically employed. The Gwet’s AC was interpreted based on the scale described by Altman [32]. Furthermore, statistical analysis was performed using binomial testing to investigate the probability of cases belonging in the first category for each scoring criterion and reviewer. The significance level for all the abovementioned statical analyses was set at 0.05.

## Results

### Patient data and characterization of lesions

Out of a total of 40 patients, 11 received a [^68^Ga]Ga-PSMA PET scan resulting in 33 lesions. The remaining 29 patients received a [^68^Ga]Ga-DOTATOC PET scan with 122 identified tumor lesions. This resulted in a total of 155 lesions. Of those lesions 65 lesions were classified as small, 75 as medium and 15 as large volume size. Additionally, 109, 11, and 35 of the 155 lesions were soft tissue, lung and bone lesions respectively.

### Noise levels

**Fig. 2:**
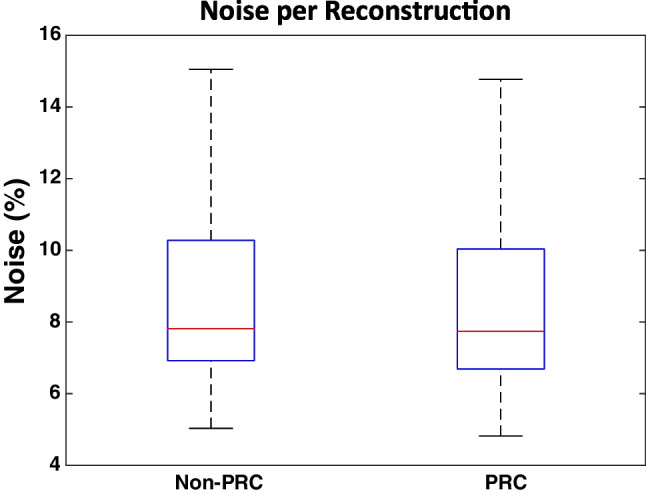
The noise levels of the PRC and non-PRC reconstructions

Liver noise levels for the PRC and the non-PRC images are presented in Fig. 2. Liver noise levels showed no statistically significant differences (p = 0.07) for PRC images (median: 7.7%, range: 4.8%−14.8%) compared to the non-PRC images (median: 7.8%, range: 5.0%−15.1%).

### Quantitative analysis

**Fig. 3:**
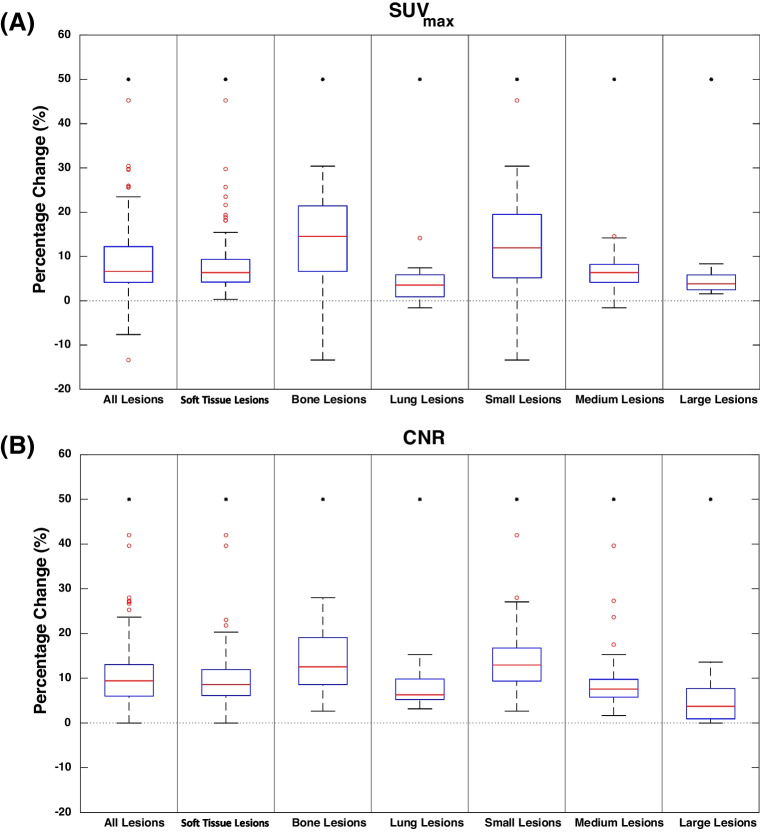
The percentage change (%) of **(A)** SUV_max_, and **(B)** CNR for the PRC versus the non-PRC image reconstructions. The asterisk (*) depicts a statistically significant difference (p < 0.001) for all cases except for lung lesions which demonstrated p = 0.010 for SUV_max_ and p = 0.001 for CNR. Categorization of lesion sizes: small (volume ≤ 1 cm^3^), medium (1 cm^3^ < volume ≤ 10 cm^3^), large (volume > 10 cm.^3^)

**Fig. 4:**
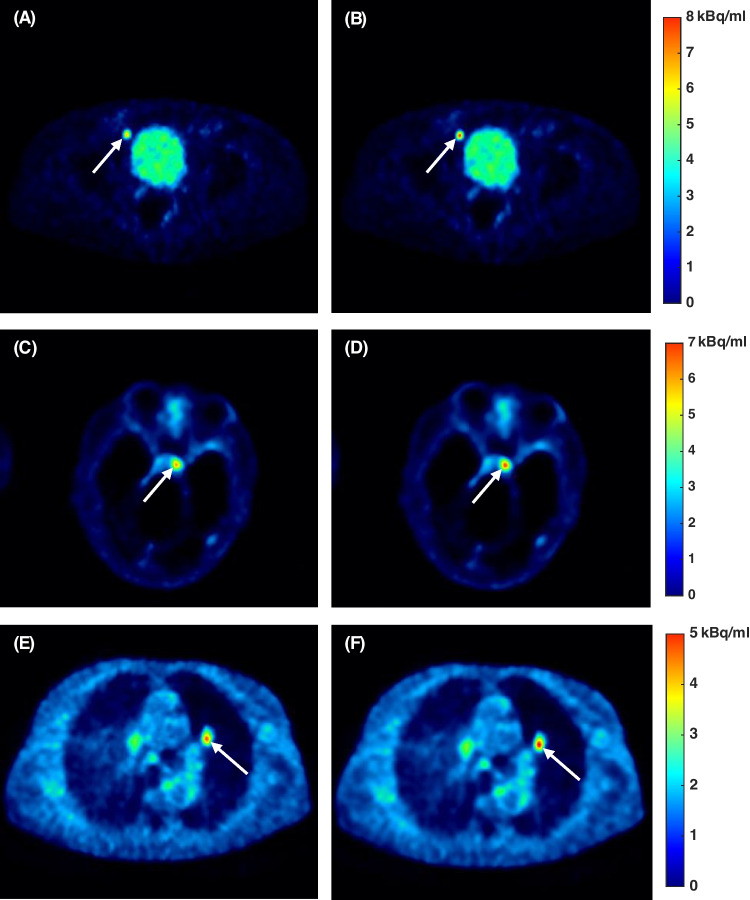
Image comparison of patient data. Soft tissue lesion (lymph node) in [^68^Ga]Ga-PSMA PET: **(A)** non-PRC, **(B)** PRC. Bone lesion in [^68^Ga]Ga-DOTATOC PET: **(C)** non-PRC, **(D)** PRC. Lung lesion in [^68^Ga]Ga-DOTATOC PET: **(E)** non-PRC, **(F)** PRC

As illustrated in Fig. 3, the PRC resulted in a systematic and statistically significant increment of the median SUV_max_ and CNR values. Increase of SUV_max_ and CNR was statistically significant for all three different lesions types with the largest increment for bone lesions (SUV_max_: 14.6%, p < 0.001; CNR: 12.5%, p < 0.001), and the lowest for lung lesions (SUV_max_: 3.6%, p = 0.010; CNR: 6.3%, p = 0.001). For soft tissue lesions, the SUV_max_ was increased by 6.4% (p < 0.001) and the CNR by 8.6% (p < 0.001). The improvements for the different lesion types were also visually notable as it is shown in Fig. 4. Moreover, the increment was demonstrated to be size-dependent. Specifically, for small lesions, the SUV_max_ and CNR were increased by 12.0% (p < 0.001) and 13.0% (p < 0.001), respectively. For the medium and large lesions, statistically significant improvements were also observed but they were smaller than 10%, with the smallest increase for the large lesions (SUV_max_: 3.8%, p < 0.001; CNR: 3.7%, p < 0.001). SUV_mean_ and SUV_peak_ demonstrated statistically significant incremental trends similar to those of SUV_max_ and CNR, as shown in Fig. S1 of the Online Resource.

### Qualitative analysis

**Fig. 5:**
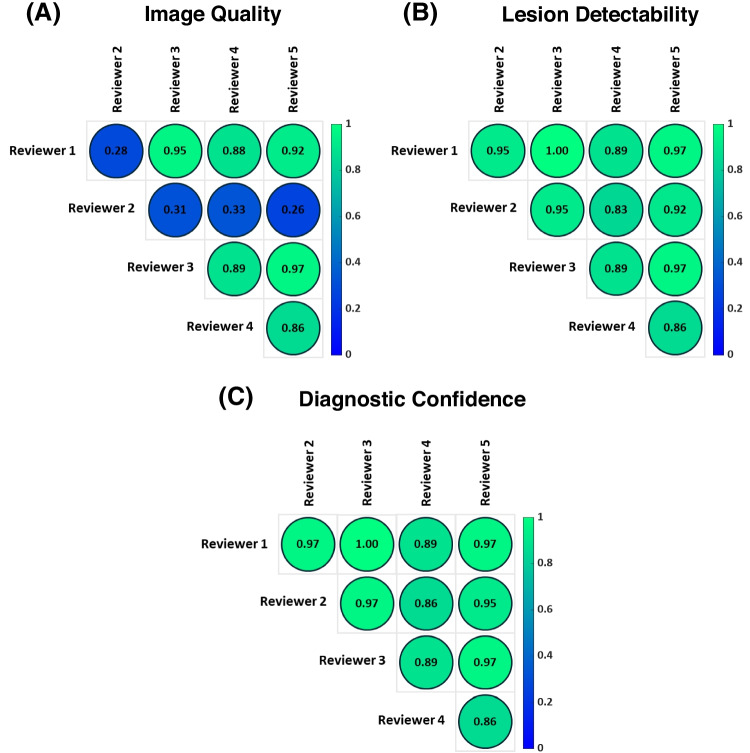
The Gwet’s AC between the different reviewers per criterion. **(A)** Image quality. **(B)** Lesion detectability. **(C)** Diagnostic confidence. The interpretation of the provided agreement coefficients is based on the scale described by Altman [32]: Poor (≤ 0.20), Fair (0.21 – 0.40), Moderate (0.41 – 0.60), Good (0.61 – 0.80), Very Good (0.81 – 1.00)

The Gwet’s AC was calculated per criterion for all the possible combinations of reviewers, and it is presented in Fig. 5. In Table 1 the overall Gwet’s AC for each criterion and the corresponding interpretation is shown. For most of the combinations, the Gwet’s AC between the different reviewers was greater than 0.81 as shown in Fig. 5. Based on the scale described by Altman [32], this can be interpreted as very good agreement (Very Good: 0.81 – 1.00). The only combinations where the agreement was low were those with the reviewer 2 for the image quality criterion. In those cases, the agreement coefficient was ranging between 0.26 and 0.33, which can be interpreted as fair (Fair: 0.21 – 0.40). The low agreement coefficients for the reviewer 2 affected also the overall Gwet’s AC for the image quality criterion as presented in Table 1. While a very good agreement was demonstrated for lesion detectability and diagnostic confidence, only a good agreement (Good: 0.61 – 0.80) was observed for the image quality.

**Table 1:** The overall Gwet’s AC alongside the 95% confidence interval (95% CI) for the different criterions. For each agreement coefficient the interpretation is provided based on the scale described by Altman [32]: Poor (≤ 0.20), Fair (0.21 – 0.40), Moderate (0.41 – 0.60), Good (0.61 – 0.80), Very Good (0.81 – 1.00)

Criterion	Gwet’s AC (95% CI)	Interpretation
Image Quality	0.71 (0.60, 0.83)	Good
Lesion Detectability	0.93 (0.87, 0.98)	Very Good
Diagnostic Confidence	0.94 (0.88, 0.99)	Very Good

Binomial testing was performed to investigate the probability of the reviewers to score the PRC reconstructed images as similar or better than the non-PRC reconstructed images for each of the three criteria. The results of this analysis are summarized in Table 2. In almost all cases, the reviewers demonstrated high probability (≥ 90%, p ≤ 0.001) to score the PRC reconstructed images as similar or better than the non-PRC reconstructed images for the studied criterions. The only exception was for reviewer 2 where the probability was 55% (p = 0.181 > 0.05) for image quality.

**Table 2:** The results of binomial testing to investigate the probability of scoring the PRC reconstructed images as similar or better than the corresponding non-PRC reconstructed images per criterion and per reviewer

Reviewers	Criterions					
	**Image Quality**		**Lesion Detectability**		**Diagnostic Confidence**	
	**Probability**	**p-value**	**Probability**	**p-value**	**Probability**	**p-value**
**Reviewer 1**	95%	< 0.001	100%	< 0.001	100%	< 0.001
**Reviewer 2**	55%	0.181	95%	< 0.001	98%	< 0.001
**Reviewer 3**	100%	< 0.001	100%	< 0.001	100%	< 0.001
**Reviewer 4**	90%	0.001	90%	0.001	90%	0.001
**Reviewer 5**	98%	< 0.001	98%	< 0.001	98%	< 0.001

## Discussion

In this study, the effect of including a tissue-independent PRC in Q.Clear reconstruction was investigated for ^68^Ga PET patient data. To allow for a fair comparison, the β value that determines the noise regularization in Q.Clear reconstructions was selected to achieve similar noise levels in PET images reconstructed with and without PRC. Compared to the non-PRC reconstructed PET images, the PRC reconstructions showed a systematic and statistically significant increase in SUV_max_ and CNR. The greatest increment was demonstrated for the bone and small lesions (≤ 1 cm^3^), whereas the smallest increase was observed for lung and large lesions (> 10 cm^3^). Additionally, PET reconstructions with PRC demonstrated better or similar image quality, lesion detectability, and diagnostic confidence compared to non-PRC reconstructions, as scored by five independent expert readers.

In clinical practice, a β value of 700 is commonly used for non-PRC reconstructions. A phantom study demonstrated that to achieve noise levels comparable to those of non-PRC reconstructions, a β value of 600 can be employed for PRC reconstructions [10]. The current study observed that using a β value of 600 for PRC reconstructions resulted in no statistically significant differences in noise levels when compared to non-PRC reconstructions with β value of 700. This indicates that phantom measurements can be used to equalize the noise levels between these different reconstruction methods. However, the median noise level of the PRC reconstructions was marginally lower compared to the corresponding non-PRC reconstructions. This implies that the use of a single β value employed for the PRC reconstructions did not result in a perfect match in noise levels for each individual patient. This minor effect was considered to be negligible compared to the variation in noise levels between the different patients also observed in the non-PRC reconstructions, see Fig. 2.

From the quantitative analysis, a systematic and statistically significant increase of SUV_max_ and CNR was observed for all types of lesions that were considered. However, this increase was limited (< 10%) for most lesions, aside from bone and small lesions (≤ 1 cm^3^). This observation that the effect of the PRC on the SUV_max_ and CNR is dependent on the lesion size is in line with previous phantom studies, where the largest improvement in ^68^Ga-PET image quality was observed for the smaller spheres [10, 24].

The qualitative analysis demonstrated that the PRC images were generally scored by expert reviewers as similar to or better than the corresponding non-PRC images for all three criteria, as shown in Table 2. The experts preferred the PRC reconstructions in all cases with small lesions. This observation is in line with the quantitative results, where the largest improvements (> 10%) in SUV_max_ and CNR were obtained for the small lesions (see Fig. 3). Additionally, the overall Gwet's AC was high for the lesion detectability and diagnostic confidence criteria demonstrating very good agreement, while good agreement was observed for image quality, see Table 1. Furthermore, very good agreement was observed between the different reviewers in the majority of the cases, see Fig. 5. Only for the image quality criterion one of the experts (reviewer 2) had mixed results, as it is shown in Table 2 and Fig. 5. Those mixed results had a diminishing effect on the overall Gwet’s AC of the image quality criterion.

An explanation for the deviation of the reviewer 2 is that the assessment process is subject to personal preferences. Beyond the three studied criteria, the reviewers were not given additional instructions on how to assess the images. Upon inquiry, the four in agreement reviewers showed a preference for higher CNR characteristics, whereas the deviating reviewer had a strong preference for less smoothing. As previously mentioned, the use of a single β value had a small but patient-varying effect on the overall image noise. In some cases the PRC resulted in slightly lower noise levels than the non-PRC, while in others the opposite was observed. This fluctuation in the image noise across different patients contributed to the results of the deviating reviewer. This clearly illustrates the fact that visual interpretation of image quality is highly subjective, and optimal reconstruction parameters, such as the β value in Q.Clear reconstructions, can depend on the experts’ preferences [33–36].

There were a few cases where the non-PRC images were preferred over the PRC images, as shown in Table 2. Those few instances were not consistent across the different experts, indicating also here the presence of the personal preference of the experts. Those instances contained predominately large or medium sized lesions for which the difference between PRC and non-PRC images was shown to be small.

In this study, Gwet's AC was employed to investigate the degree of agreement between the reviewers. While kappa statistics are commonly used in studies exploring inter-reviewer agreement, Gwet's AC is more stable when there is high prevalence [29–31]. High prevalence can lead to high absolute percentage of observer agreement coupled with low kappa values [29–31]. The qualitative data in this study featured high prevalence, since the reviewers predominantly scored the PRC images as similar to or better than the non-PRC images. Therefore, due to this nature of our data, Gwet’s AC was selected as the preferred agreement measurement.

A limitation of the current study is that the PRC method is a tissue-independent technique based on the positron range distribution profile of ^68^Ga in water medium [10]. As a result, the used PRC is appropriate for soft tissue lesions. However, simulated phantom data showed that a tissue-independent PRC was over-correcting the bone tissue and under-correcting the lung tissue, although the image quality in general was improved [20, 22]. Thus, the impact of PRC on the SUV_max_ and CNR of bone and lung lesions presented in this study is also determined by over- and under-correction, respectively. In the literature, more advanced techniques have been proposed that consider different tissue types and tissue boundaries and are generally regarded as more accurate than a tissue-independent PRC [21, 22, 24]. In those cases, the information regarding the tissue structure is typically determined through segmentation of the accompanying magnetic resonance imaging (MRI) or computed tomography (CT) data. However, due to this dependency on underlying tissue information, those PRCs are prone to misalignments between the PET and the corresponding CT/MRI data caused by patient motion. Notorious areas for respiratory motion artifacts are the upper abdominal organs and basal lungs. Such misalignments can potentially be mitigated via inclusion of respiratory motion correction techniques in the reconstruction [37–39]. Additionally, our proposed method, although straightforward since it is tissue-independent, demonstrated added clinical value in the current study while increasing the reconstruction time by less than 5%. As such, a tissue-independent PRC reconstruction could already be presented to the nuclear medicine physicians next to a standard non-PRC reconstruction to improve lesion detectability and diagnostic confidence. Meanwhile, more advanced PRC models, such as tissue-dependent PRC, can be translated into a clinical setting.

Finally, for lesion delineation a 50% isocontour thresholding technique without background correction was applied to the PRC images. Next, the obtained VOIs were mapped to the non-PRC images. We acknowledge the fact that this technique has limitations with respect to delineation accuracy and does not represent clinical practice in which lesion delineation would be performed onto each image separately. However, the focus of the current study is to demonstrate the impact of PRC on clinical ^68^Ga PET images, instead of achieving the most accurate lesion delineation. Moreover, as the PRC results in increased lesion SUV values, this will have a direct effect on the delineated lesion volume, SUV_mean_, SUV_peak_, and CNR values and may thereby complicate the comparison. Therefore, in order to achieve a fair comparison in lesion quantification between the PRC and non-PRC images identical lesion VOIs were chosen for both images.

## Conclusion

The application of a tissue-independent ^68^Ga-specific PRC during PET reconstructions resulted in a positive impact on the SUV_max_ and CNR of the different lesion types, with a higher impact on smaller lesions. Additionally, although visual assessment of patient data was demonstrated to be subjective considering the preference of the reader, the ^68^Ga-PET images reconstructed with PRC were scored to have similar or better image quality, lesion detectability, and diagnostic confidence compared to the ^68^Ga-PET images reconstructed without PRC.

## Supplementary Information

Below is the link to the electronic supplementary material.

Supplementary file1 (DOCX 43.7 KB)

## Data Availability

The datasets generated during and/or analysed during the current study are available from the corresponding author on reasonable request.
