# Supplementary material for: Impact of tissue-independent positron range correction on [68Ga]Ga-DOTATOC and [68Ga]Ga-PSMA PET image reconstructions: a patient data study
Source: Eur J Nucl Med Mol Imaging. 2025 Jan 29;52(7):2538–48. doi: 10.1007/s00259-024-07061-6 (PMC12119741; doi:10.1007/s00259-024-07061-6)
Supplement: Supplementary file 1 — Supplementary file1 (DOCX 43.7 KB) [file 259_2024_7061_MOESM1_ESM.docx]

**Electronic Supplementary Material (ESM)**

**Title:** Impact of tissue-independent positron range correction on [^68^Ga]Ga-DOTATOC and [^68^Ga]Ga-PSMA PET image reconstructions: a patient data study

**Journal:** European Journal of Nuclear Medicine and Molecular Imaging

**Authors:** Prodromos Gavriilidis^1,2,3^, Felix M. Mottaghy^4,1^, Michel Koole^3^, Tineke van de Weijer^1,6^, Cristina Mitea^1,2^, Jochem A.J. van der Pol^1,7^, Thiemo J.A. van Nijnatten^1^^,2^, Floris P. Jansen^5^, Roel Wierts^1,*^

**Affiliations:**

^1^ Department of Radiology and Nuclear Medicine, Maastricht University Medical Center, Maastricht, The Netherlands,

^2^ Research Institute for Oncology and Reproduction (GROW), Maastricht University, Maastricht, The Netherlands,

^3^ Nuclear Medicine and Molecular Imaging, Department of Imaging & Pathology, KU Leuven, Leuven, Belgium,

^4^ Department of Nuclear Medicine, RWTH University Hospital, Aachen, Germany,

^5^ Molecular Imaging, GE HealthCare, Waukesha, Wisconsin, USA,

^6^ Research Institute of Nutrition Translational Research in Metabolism (NUTRIM), Maastricht University, Maastricht, The Netherlands,

^7^ Cardiovascular Research Institute Maastricht (CARIM), Maastricht University, Maastricht, The Netherlands.

^*^ Corresponding Author, e-mail address: roel.wierts@mumc.nl

**Quantitative analysis: SUV_mean_ and SUV_peak_**

As shown in Fig. S1 of the supplementary material, the PRC resulted in a systematic and statistically significant increase in the median SUV_mean_ and SUV_peak_. Among the three different types of lesions, bone lesions demonstrated the largest increment (SUV_mean_: 13.1%, p < 0.001; SUV_peak_: 10.5%, p < 0.001). The SUV_mean_ and SUV_peak_ were also increased for soft tissue lesions by 5.6% (p < 0.001) and 6.0% (p < 0.001), respectively. The lowest increase among the different lesion types was observed in lung lesions (SUV_mean_: 4.7%, p = 0.001; SUV_peak_: 5.0%, p = 0.001). The effect on SUV_mean_ and SUV_peak_ was also size-dependent. In small lesions, the SUV_mean_ was increased by 11.7% (p < 0.001) and the SUV_peak_ by 9.1% (p < 0.001), while medium and large lesions demonstrated smaller improvements. The smallest increase was observed in large lesions (SUV_mean_: 2.4%, p < 0.001; SUV_peak_: 3.7%, p < 0.001).

**Fig. S1:** The percentage change (%) of **(A)** SUV_mean_, and **(B)** SUV_peak_ for the PRC versus the non-PRC image reconstructions. The asterisk (*) depicts a statistically significant difference (p < 0.001) for all cases except for lung lesions which demonstrated p = 0.001 for both SUV_mean_ and SUV_peak_. Categorization of lesion sizes: small (volume ≤ 1 cm^3^), medium (1 cm^3^ < volume ≤ 10 cm^3^), large (volume > 10 cm^3^).
